# Pulmonary dendritic cells and alveolar macrophages are regulated by γδ T cells during the resolution of *S. pneumoniae*-induced inflammation

**DOI:** 10.1002/path.2149

**Published:** 2007-05

**Authors:** AC Kirby, DJ Newton, SR Carding, PM Kaye

**Affiliations:** 1 Immunology and Infection Unit, Department of Biology, University of York and Hull York Medical SchoolUK; 2 Research Institute for Molecular and Cellular Biology, University of LeedsLeeds, UK

**Keywords:** γδ T cells, dendritic cells, alveolar macrophages, inflammation, resolution

## Abstract

γδ T cells commonly associate with mucosal and epithelial sites, fulfilling a variety of immunoregulatory functions. While lung γδ T cells have well-characterized pro-inflammatory activity, their potential role in the resolution of lung inflammation has yet to be explored in any detail. Indeed, given the importance of minimizing inflammation, the cellular mechanisms driving the resolution of lung inflammation are poorly understood. Using a murine model of acute *Streptococcus pneumoniae*-mediated lung inflammation, we now show that resolution of inflammation following bacterial clearance is associated with a > 30-fold increase in γδ T-cell number. Although inflammation eventually resolves in TCRδ^−/−^ mice, elevated numbers of alveolar macrophages and pulmonary dendritic cells, and the appearance of well-formed granulomas in lungs of TCRδ^−/−^ mice, together indicated a role for γδ T cells in regulating mononuclear phagocyte number. *Ex vivo*, both alveolar macrophages and pulmonary dendritic cells were susceptible to lung γδ T cell-mediated cytotoxicity, the first demonstration of such activity against a dendritic cell population. These findings support a model whereby expansion of γδ T cells helps restore mononuclear phagocyte numbers to homeostatic levels, protecting the lung from the consequences of inappropriate inflammation. Copyright © 2007 Pathological Society of Great Britain and Ireland. Published by John Wiley & Sons, Ltd.

## Introduction

Appropriate regulation and resolution of acute pulmonary inflammatory responses are critical for maintenance of tissue integrity, avoidance of host-mediated pathology, and host survival. Lung inflammation following pneumococcal infection involves extensive inflammatory cell influx into tissues and alveolar spaces, perivascular oedema, and substantial consolidation 1,2. Nevertheless, following bacterial clearance, inflammation rapidly resolves, leaving little structural or morphological evidence to indicate that the response occurred 2,3. However, the mechanisms involved in resolving inflammation are not currently well understood.

A major cell population involved in regulating pulmonary innate immune responses is the alveolar macrophage (AM). AMs control the threshold at which innate responses to streptococcal infection are initiated 4 and are involved in resolution of acute pulmonary inflammation through phagocytic clearance of apoptotic neutrophils 5–7. Moreover, apoptotic AMs may reduce inflammatory responses and are themselves cleared by AMs 8. While the influx of new AMs continues after bacterial clearance 9, most likely to remove inflammatory ‘debris’, the question of how AMs are themselves regulated during resolution remains open.

A recently described mechanism for control of macrophage responses is through the direct action of γδ T cells 10,11. Killing of activated macrophages by γδ T cells prevents excessive inflammatory res- ponses and necrotic liver damage following *Listeria* infection 12, and restricts parasite growth and host-mediated pathology in *Toxoplasma gondii* infection 11. Finally, lung γδ T cells isolated from *Mycobacterium bovis* BCG-infected mice are cytotoxic against infected peritoneal macrophages 13. However, γδ T cells are a rare population in most tissues 14 and the extent to which γδ T cells mediate resolution of inflammation following pathogen clearance remains unclear.

While a role for γδ T cells in *Streptococcus pneumoniae* infection has not previously been described, γδ T cells have various immunoregulatory functions in other pulmonary inflammation models. γδ T-cell responses appear to be responsible for appropriate cytokine responses to influenza 15,16 and *Klebsiella pneumoniae* 17; may influence the influx of inflammatory cells to control *Nocardia asteroides* infection 18,19; and contribute to IFNγ 13 and IL-17 20 production following mycobacterial challenge. In contrast, γδ T cells may inhibit pro-inflammatory responses to *Cryptococcus neoformans*, possibly preventing excessive Th1 responses 21. However, in none of these models has the role of γδ T cells during the resolution phase of the response been examined.

We have previously described a model of *S. pneumoniae*-induced lung inflammation in which an acute, phagocyte-dominated inflammatory response results in pathogen clearance within 5 days 2. Intranasal (i.n.) challenge with *S. pneumoniae* serotype 6B induces inflammation which results in substantial modulation of AM and pulmonary dendritic cell (pulDC) populations which extends well beyond pathogen clearance. Here we demonstrate that the resolution phase of *S. pneumoniae*-induced pulmonary inflammation is accompanied by a more than 30-fold increase in lung γδ T-cell number. Nevertheless, these cells do not contribute to bacterial clearance, but are directly cytotoxic against AMs and pulDCs. Together with the appearance of small coherent granulomas in TCRδ^−/−^ mice, these studies suggest that cytotoxic γδ T cells play an important role in regulating the expansion and homeostatic control of these inflammatory cell populations.

## Materials and methods

### Mice and pneumococcal infection

C57BL/6 and B6.TCRδ^−/−^ mice were bred and housed under barrier conditions at LSHTM and the University of York, and supplied with food and water *ad libitum*. Mice were infected i.n. with 10^8^–10^9^ cfu of a clinical isolate of *S. pneumoniae* serotype 6B (Microbiology Department, Royal Free Hospital) as previously described 2,9. Animal experimentation was performed with LSHTM and University of York Animal Procedures Ethics Committee and UK Home Office approval.

### Tissue preparation

Recovery of bronchoalveolar lavage fluid (BALF) and preparation of whole lungs (no BALF harvest) were performed as previously described 2,9. Viable cell counts were determined by trypan blue exclusion. Tissue sections were prepared and imaged as previously described 9.

### Flow cytometry and cell sorting

Flow cytometry was carried out using the following clones and reagents (all BD Pharmingen): GL3 (TCRδ); 145.2C11 (CD3); H57-597 (TCR β); GK1.5 (CD4);, 53-6.7 (CD8α); RA3.6B2 (B220); HL3 (CD11c); 2G9 (MHC-II); and M1/70 (CD11b), together with appropriate isotype controls. All samples were treated with anti-Fcγ RII/III (2.4G2) prior to specific staining. Samples were acquired on a FACSCalibur™ flow cytometer and analysed with CellQuest Pro™ software (both Becton Dickinson Oxford, UK), or on a Cyan flow cytometer and analysed with Summit v4.1 software (both DakoCytomation).

CD11c^+^ AMs and pulDCs were sorted from whole lung cell preparations following MACS (Miltenyi Biotech, Germany) enrichment 9 using a MoFlo cell sorter (DakoCytomation). CD3^+^ T-cell populations from whole lung cells or CD11c-depleted lung cells were enriched by MACS. Depletion of αβ T cells from enriched CD3^+^ cells was also performed by MACS.

### Cytotoxic assay

Cytotoxic activity of CD3^+^ cells, or CD3^+^ cells depleted of αβ^+^ T cells, was assessed using a Live/Dead cell-mediated cytotoxicity kit (Molecular Probes) according to the manufacturer's instructions and established protocols 10. Briefly, sorted, DiOC-labelled AMs or pulDCs (1 × 10^5^ per well) were used as targets in triplicate cultures incubated with unlabelled effector cells in the presence of propidium iodide (PI) for 4 h at 37 °C. Killing was assessed by flow cytometry, with dead targets being DiOC^+^PI^+^. Specific killing was determined in comparison with wells containing targets only. F(ab)_2_ fragments of GL3 (anti-TCRδ) or control antibody were included at 10 µg/ml in some assays.

## Results

### Pulmonary γδ T-cell accumulation associates with resolution of inflammation

**Figure 1:**
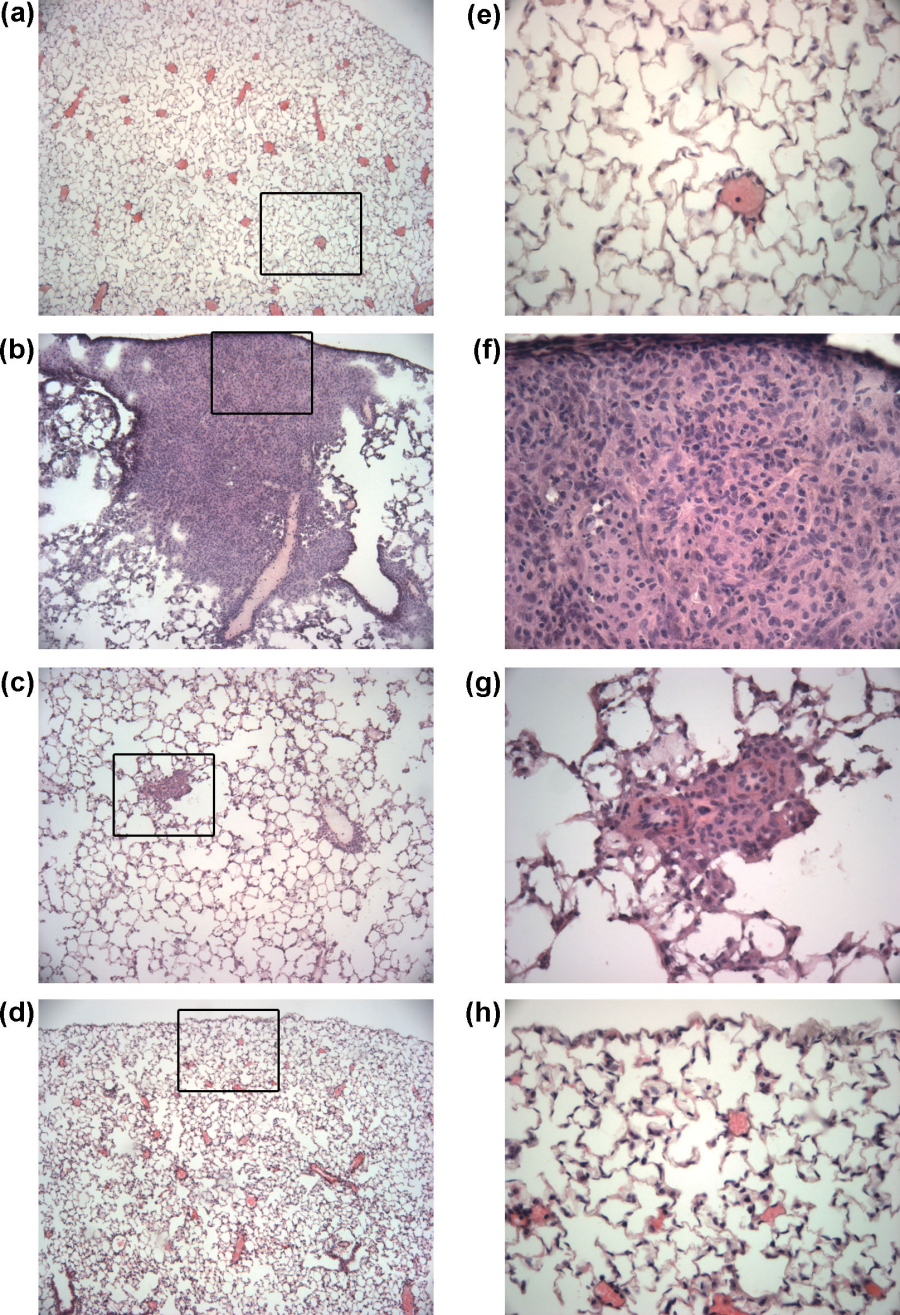
Inflammation and resolution within lung tissue following *S. pneumoniae* serotype 6B challenge. Haematoxylin and eosin staining of lung sections from (a, e) naïve mice or from mice at (b, f) 3 days, (c, g) 7 days, and (d, h) 14 days post-*S. pneumoniae* challenge (*n* = 6–12 at each time point). Original magnifications: (a–d) × 100; (e–h) × 400. The boxes in a–d indicate the area of image presented at higher magnification

Intranasal (i.n.) challenge with non-lethal *S. pneumoniae* serotype 6B induces a rapid, transient neutrophil-dominated phagocyte response; bacterial clearance by day 5; and a resolution phase extending to more than 14 days post-challenge 2. Early pathological features are typical of pneumonia, with inflammatory cell migration into alveoli resulting in extensive consolidation (Figures 1a and 1b). Nevertheless, this consolidation rapidly resolves after the initial pathogen insult is cleared, with only small inflammatory foci remaining by day 7 (Figure 1c). By 14 days, the challenged lung is largely histopathologically indistinguishable from that of unchallenged mice (Figure 1d).

**Figure 2:**
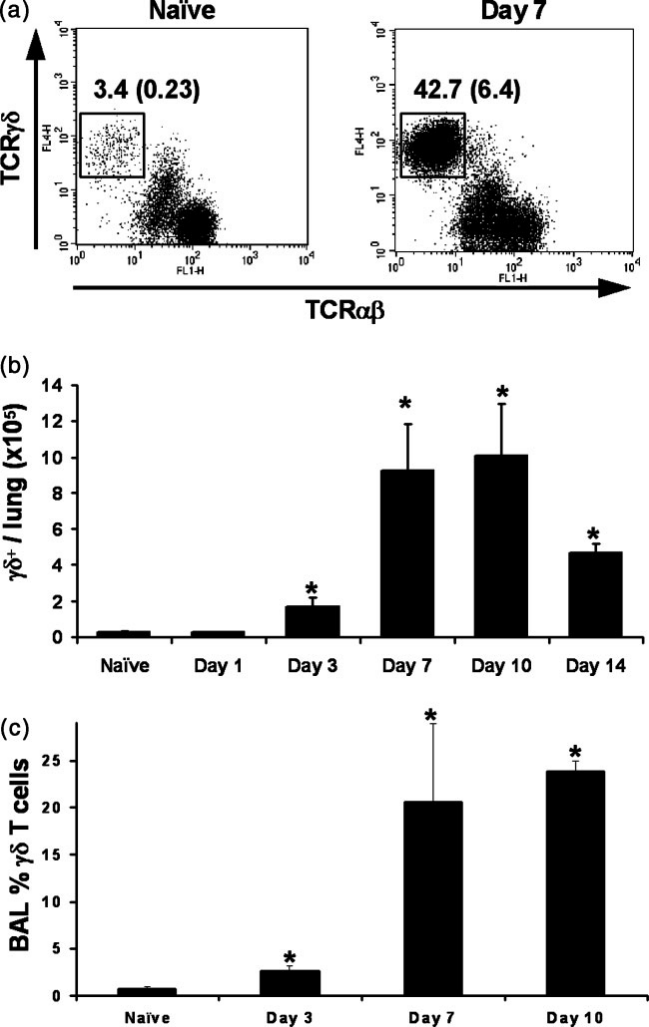
γδ T-cell number in the lung following pneumococcal challenge. (a) Gated CD3^+^ lung cells were compared for expression of TCRδ and TCRβ at day 0 (naïve) and day 7 following *S. pneumoniae* challenge. Numbers represent TCRδ^+^ cells as a percentage of CD3^+^ cells and of total lung cells (in parentheses). Data are representative of at least 12 mice. (b) The absolute number of γδ T cells in the total lung and (c) the % γδ T cells within BALF at the indicated times following *S. pneumoniae* challenge are shown. Graph points represent mean ( ± 1 SD). All graphs represent 6–18 mice at each time point. *p < 0.05 versus naïve control, by Student's *t*-test

Since CD4^+^ 22 and CD8^+^αβ T cells 23, as well as γδ T cells 24, have been ascribed immunoregulatory properties in the inflamed lung, we investigated whether these populations were involved in the rapid restoration of consolidated lung tissue back to an uninflamed state. Therefore, lung lymphocyte populations were analysed during the initiation and resolution phases of *S. pneumoniae*-induced inflammation. Significant but slight (less than two-fold) changes in the numbers of pulmonary CD4^+^ or CD8^+^ T cells, B cells or NK cells were observed over the 14 days following challenge (data not shown). In contrast, absolute numbers of γδ T cells in the lung following *S. pneumoniae* challenge were increased more than 30-fold at the peak of the response (days 7–10; Figure 2).

In naïve animals, CD3^+^TCRβ^−^TCRγδ^+^γδ T cells (Figure 2a) comprised 3.03 ± 1.2 × 10^4^ cells per lung (Figure 2b), in agreement with recent estimates 25. While this number was significantly increased as early as day 3 post-*S. pneumoniae* challenge (*p* < 0.05), the response peaked at days 7–10 (Figure 2b), despite there being no detectable bacteria in the lung later than day 5 post-challenge (refs 2 and 9 and data not shown). Increased γδ T-cell populations were strikingly restricted to the lung, with less than two-fold changes in the proportions of γδ T cells observed in draining lymph nodes, blood, and spleen of infected mice (data not shown).

*S. pneumoniae* challenge results in large numbers of inflammatory cells entering alveoli (Figure 1b) 26. We examined BALF from naïve and *S. pneumoniae*-challenged mice to determine whether γδ T cells access this compartment. Although BALF from naïve mice contained few lymphocytes, a low percentage expressed the γδ TCR (0.7 ± 0.2%, Figure 2c). Following pneumococcal challenge, TCRγδ^+^ cells increased in BALF in correlation with their increase in total lung, comprising 23.8 ± 1.2% of BALF cells at day 10 (Figure 2c). Thus, *S. pneumoniae* challenge induces a strong pulmonary γδ T-cell response, with cells entering alveolar spaces as well as lung tissue. Notably, the peak γδ T-cell response occurs after bacterial clearance.

### AM and pulDC responses are dysregulated in the absence of γδ T cells

In wild-type mice, significant characteristics of the resolution phase following clearance of *S. pneumoniae* serotype 6B are the changes which occur within AM and pulDC populations 2,9. Therefore, AMs and pulDCs were examined in wild-type and γδ T-cell-deficient (TCRδ^−/−^) mice at days 7–14 post-challenge, covering the peak of the γδ T-cell response during resolution. TCRδ^−/−^ mice given *S. pneumoniae* serotype 6B did not succumb to infection and no bacteria were recovered from the lungs of TCRδ^−/−^ mice at days 7–14 post-challenge (*n* = 24).

**Figure 3:**
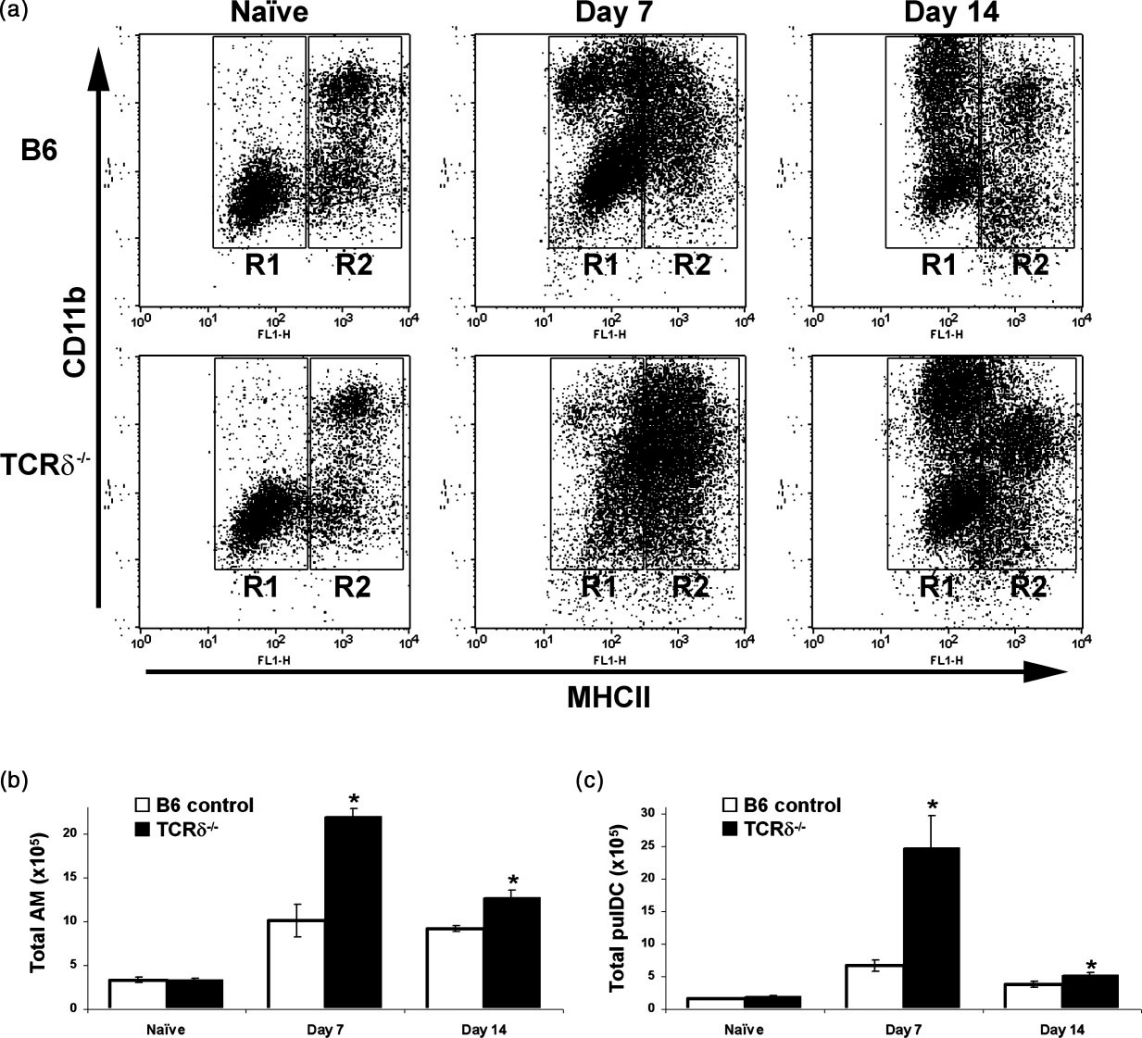
Dysregulation of AM and pulDC responses in TCRδ^−/−^ mice. Total lung cells from na*ï*ve mice, or mice challenged with *S. pneumoniae*, were analysed for AMs and pulDCs. (a) Gated CD11c^+^ lung cells in wild-type (upper plots) and TCRδ^−/−^ (lower plots) mice analysed for expression of MHCII (*x*-axis) and CD11b (*y*-axis). R1 defines AMs and R2 defines pulDCs in na*ï*ve mice. During infection, additional flow cytometric parameters are used to conclusively identify AMs and pulDCs, as described elsewhere 9. (b) Absolute numbers of AMs and (c) pulDCs in the lungs of wild-type and TCRδ^−/−^ mice before and following *S. pneumoniae* challenge (*n* = 6 or more at each time point). Bars represent mean ( ± 1 SD). *p < 0.05 versus wild-type control at the same time point, by Student's *t*-test

AMs and pulDCs were identified in parallel based on differential expression of CD11c, MHCII, CD11b, and on size, granularity, and autofluorescence 9. Naïve wild-type and TCRδ^−/−^ mice had qualitatively and quantitatively comparable populations of CD11c^HI^MHCII^INT^CD11b^LO^ AMs and CD11c^HI^MHCII^HI^CD11b^+/−^ pulDCs (Figure 3a, left panels). *S. pneumoniae* challenge of wild-type mice induced phenotypic and quantitative changes in both populations, including the appearance of CD11b^HI^ AMs 9 (Figure 3a, upper panels). At day 7 post-challenge, both AMs (*p* < 0.001) and pulDCs (*p* < 0.001) were significantly elevated in number compared with naïve controls. Both AM and pulDC numbers remained elevated at day 14 post-challenge, although pulDCs were significantly reduced in number compared with day 7 (*p* = 0.002; Figure 3c).

Analysis of TCRδ^−/−^ mice revealed exaggerated AM and pulDC responses, with consistent increases in CD11b^+^ cells compared with wild-type controls (Figure 3a). In TCRδ^−/−^ mice, AMs were two-fold more abundant than in wild-type mice at day 7 post-challenge (*p* < 0.001) and remained significantly above control numbers at day 14 (*p* < 0.001; Figure 3b). Notably, CD11b^HI^, newly influxed AMs 9 were significantly increased in TCRδ^−/−^ mice both at day 7 (Figure 3a and data not shown; mean 8.5 ± 2.6 × 10^5^) compared with wild-type mice (4.1 ± 2.0 × 10^5^; *p* < 0.01) and at day 14 (6.5 ± 1.6 × 10^5^ versus 4.1 ± 0.5 × 10^5^; *p* < 0.0001).

Similarly, pulDC responses in TCRδ^−/−^ mice were significantly increased, being more than three-fold more abundant than controls at day 7 (*p* < 0.001), remaining elevated at day 14 (*p* = 0.02; Figure 3c). In contrast to AMs, influxing pulDCs in both TCRδ^−/−^ and control mice were predominantly CD11b^HI^ (Figure 3a) 9. These data suggest γδ T-cell involvement in regulating AM and pulDC numbers during resolution of *S. pneumoniae*-mediated inflammation.

### γδ T cells kill both AMs and pulDCs

Since activated splenic and peritoneal macrophages are susceptible to TCRδ-dependent, γδ T-cell-mediated killing 10–12, we examined whether γδ T cells might exert cytotoxic activity against AMs and pulDCs following pneumococcal challenge. Purified (>95%) AMs or pulDCs were co-cultured with MACS-purified CD3^+^ cells from CD11c^+^-depleted day 7 post-challenge lung cell suspensions, of which 25–40% were TCRδ^+^ cells. Lung populations depleted of αβ T cells were also used, in which effector CD3^+^ cells were more than 95% TCRγδ^+^.

**Figure 4:**
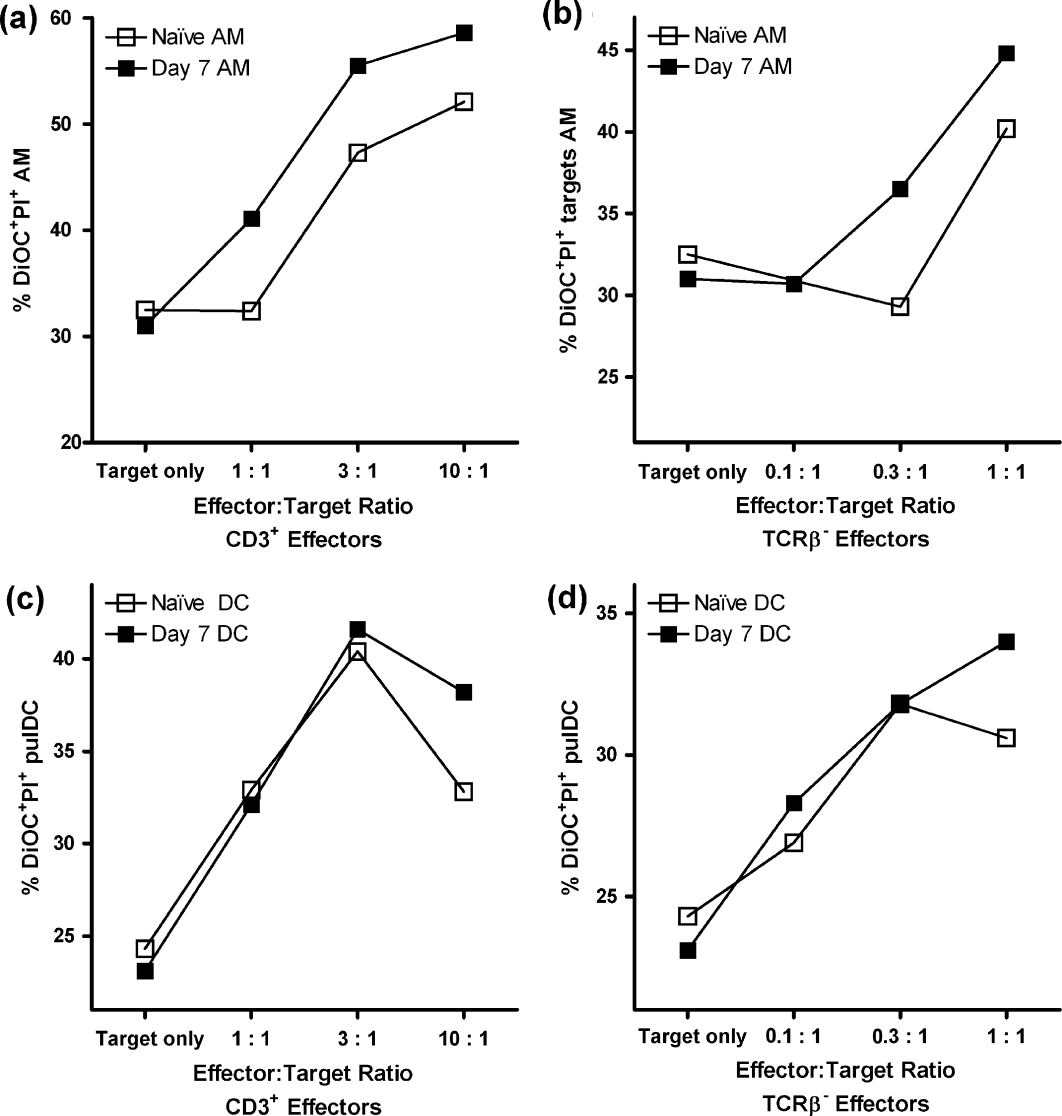
γδ T cells are cytotoxic for AMs and pulDCs. Enriched lung T cells from day 7 following *S. pneumoniae* challenge were used in various ratios as effectors against DiOC-labelled AM (a, b) and (c, d) pulDC targets in a cytotoxic assay. The percentage of dead targets, identified as DiOC^+^PI^+^ cells, is shown. Total CD3^+^ (a, c) or TCRβ-depleted CD3^+^ (b, d) cells used as effectors against labelled targets from na*ï*ve (□) or infected (▪) mice. At least 10 000 DiOC^+^ target cells were analysed in each case. Plots are representative of four separate experiments

CD3^+^ effector cells caused dose-dependent death of AMs taken from lungs at day 7 post-challenge (Figure 4a), as did αβ T-cell-depleted, enriched γδ T cells (Figure 4b). Both total CD3^+^ and αβ T-cell-depleted effector cells were also efficient in killing pulDCs from mice at day 7 post-challenge (Figures 4c and 4d). Furthermore, inclusion of blocking antibody against TCRδ, but not control antibody, reduced pulDC killing by CD3^+^ effectors at a 3 : 1 effector : target ratio by 52 ± 4% (*p* = 0.019).

Previous studies demonstrated that only activated peritoneal macrophages were effectively killed by γδ T cells 10. We examined whether AMs and pulDCs from naïve mice could act as targets for γδ T cells. Both CD3^+^ and γδ T-cell-enriched effectors from day 7 lungs killed naïve AMs, although less efficiently than AMs from *S. pneumoniae*-challenged mice (Figures 4a and 4b). Surprisingly, naïve pulDCs were equally susceptible to killing, as were those pulDCs from challenged hosts (Figures 4c and 4d). These data represent the first observation of γδ T-cell-mediated killing of a dendritic cell population.

### γδ T-cell deficiency results in granuloma development during resolution of *S. pneumoniae*-induced inflammation

To examine whether dysregulation of AMs and pulDCs affected the histopathological features of the resolving lung, we examined wild-type and TCRδ^−/−^ mice at day 5 post-challenge. At this stage, while resolution is under way, significant areas of inflammation remain for histological evaluation.

**Figure 5:**
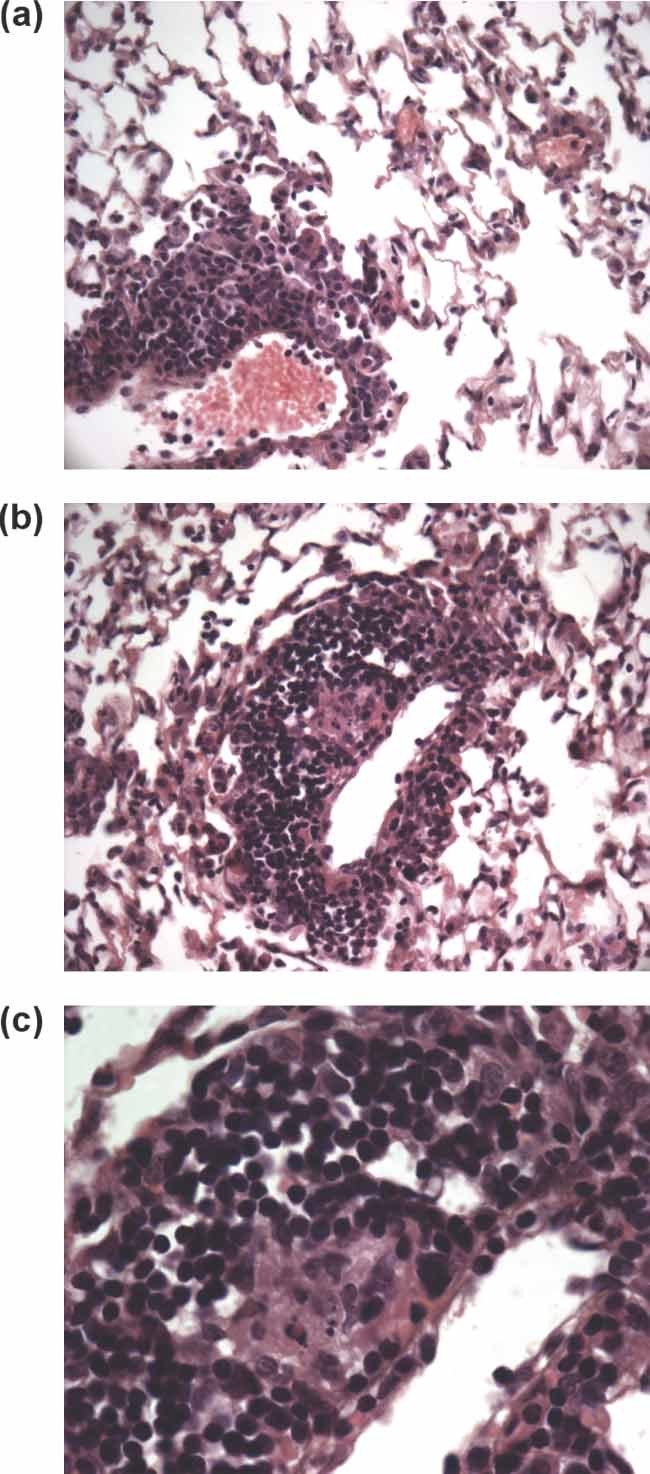
Altered histopathological features during resolution in TCRδ^−/−^ mice. Haematoxylin and eosin-stained sections of lung tissue from (a) wild-type and (b, c) TCRδ^−/−^ mice challenged 5 days previously with *S. pneumoniae* 6B. Original magnification: (a, b) × 400; (c) × 1000

Wild-type lungs at day 5 following pneumococcal challenge showed relatively small areas of predominantly perivascular inflammation remaining throughout the tissue (Figure 5a). In TCRδ^−/−^ mice, perivascular inflammation was also apparent at day 5. However, TCRδ^−/−^ lungs also exhibited small granulomas within inflammatory foci (Figures 5b and 5c). A total of 27 granulomas were present in 48 inflammatory foci at day 5 in the TCRδ^−/−^ group (*n* = 4), compared with no granulomas from 76 foci in the control group (*n* = 4). No other differences in histopathological features were apparent between the groups. In both wild-type and TCRδ^−/−^ mice, all coherent inflammatory foci and any associated granulomas had resolved by day 14 post-challenge (data not shown).

## Discussion

The present study uses a model of pneumococcal challenge to examine possible mechanisms underlying the resolution of inflammation following pathogen clearance. *S. pneumoniae* challenge induces a substantial γδ T-cell response which does not appear to contribute to antibacterial activity. γδ T cells may most commonly facilitate effective responses to pulmonary and non-pulmonary pathogens 17–19,21,26,27, and absence of γδ T cells may profoundly influence host survival 19. In contrast, TCRδ^−/−^ mice have no deficiency in clearance of *S. pneumoniae* and the γδ T-cell response peaks after bacterial clearance, reflecting earlier studies of viral lung infections 15,28. Ongoing studies indicate that multiple γδ T-cell subsets, including the Vγ1 and Vγ4 subsets recently observed in naïve lung 25, are involved following pneumococcal challenge (Kirby *et al*, unpublished data). While both Vγ1 and Vγ4 subsets are also involved in early responses to mycobacteria 13, whether γδ T-cell subsets have differential functions during bacterial lung infections remains to be defined.

Absence of γδ T cells results in striking quantitative increases in AMs and pulDCs during resolution of *S. pneumoniae*-induced inflammation, including notably elevated numbers of CD11b^HI^ AMs. CD11b expression is required for AM influx following pneumococcal challenge 9, suggesting that γδ T-cell deficiency leads to excessive accumulation of newly recruited AMs. In the wild-type host, γδ T cells may down-regulate local production of inflammatory mediators, limiting AM and pulDC recruitment. However, our data strongly suggest that γδ T-cell regulatory activity is exerted via direct cytotoxicity. In other lung infection models, γδ T-cell cytotoxicity is not a consistent phenotype. While γδ T cells from early *M. bovis* infection were cytotoxic 13, those from influenza-infected mice lacked cytotoxic activity 29. These differences may reflect the functional heterogeneity of γδ T-cell populations and their ability to respond to differing inflammatory environments 30. Importantly, however, rather than contributing to pathogen clearance, as in *T. gondii* 11 and *M. bovis* 13 infections, γδ T-cell cytotoxicity in the current model is clearly resolution-associated.

Strikingly, pulDCs as well as AMs are susceptible to lung γδ T-cell-mediated cytotoxicity. This is the first demonstration that γδ T cells can kill not only macrophages, but also dendritic cells. While Fas expression, involved in γδ T-cell killing of splenic macrophages 31, is increased on pulDCs, but not AMs, following pneumococcal challenge (our unpublished observations), the specific mechanism of pulDC killing remains under investigation. The role of pulmonary Vγ1^+^ cells 25, central to this process in both *Listeria* 10,32 and *Toxoplasma gondii* 11 infections, also remains undetermined.

Isolated γδ T cells have previously only been shown to kill activated, but not naïve, macrophages 10,13. Lung γδ T cells recovered 7 days after *S. pneumoniae* challenge killed both naïve and infected AMs and pulDCs. While this suggests a role for γδ T cells in regulating these populations during homeostasis, naïve lungs contain only approximately 3 × 10^4^γδ T cells, and no difference in AM or pulDC number was apparent between unchallenged wild-type and TCRδ^−/−^ mice. Although this does not formally exclude a role for γδ T cells in homeostatic regulation of AMs and pulDCs, such a function is considered unlikely. Perhaps of more interest for future study, our data suggest that both AMs and pulDCs constitutively express ligands able to direct γδ T-cell killing.

That AM and pulDC numbers eventually decline in TCRδ^−/−^ mice may suggest a non-essential contribution of γδ T cells during late resolution. However, this observation also emphasizes the extent of γδ T-cell-mediated control of AM and pulDC populations observed over days 7–14. In the current model, the progressive reduction in AMs and pulDCs in TCRδ^−/−^ mice likely reflects general reductions in inflammatory stimuli in the absence of pathogen load post-day 5. The γδ T-cell-mediated reduction of pulDC numbers once antigen load is significantly reduced (days 3–5) may restrict inappropriate antigen presentation and inflammatory mediator production. In contrast, AM recruitment following bacterial clearance 9 is required for the removal of cellular debris 7. Therefore, even in the presence of γδ T cells, the return of AM numbers to pre-challenge levels takes substantially longer than for pulDCs.

Excessive numbers of activated macrophages are not beneficial to the host 11,12,33. This study strongly indicates that γδ T cells control AM numbers during resolution of lung inflammation. Since γδ T cells kill activated tissue macrophages 10, it is possible that they also act against lung tissue macrophages in the current model. That TCRδ^−/−^ mice exhibit small granulomas within inflammatory foci is suggestive of such activity, although this has not been directly examined. Alternatively, granuloma formation may occur due to dysregulated AM and pulDC activity. Studies using mycobacterial infections have demonstrated γδ T-cell association with granulomas and influence upon their formation 34,35. γδ T cells may, therefore, help to determine the correct cellular composition of granulomas associated with bacterial infection. In the current model, spontaneous resolution of granulomas by day 14 most likely reflects the transient nature of pneumococcal-induced inflammation, as described above. However, in chronic disease, where inflammatory stimuli or pathogen load persist, γδ T cells may play a major role in regulating pathological features such as granulomas.

Together, the current data enhance the immunoregulatory reputation of γδ T cells and suggest that γδ T-cell-mediated cytotoxicity against inflammatory populations may be widespread during resolution of inflammation.
